# Recruitment of FOR20 and OFD1 onto pericentriolar satellites and centrosomes depends on the formation of a ternary complex with KIAA0753

**DOI:** 10.1186/2046-2530-4-S1-P69

**Published:** 2015-07-13

**Authors:** V Chevrier, F Lembo, S Audebert, JP Borg, D Birnbaum, O Rosnet

**Affiliations:** 1 Aix-Marseille University, Marseille, France; 2 Cancer Research Center of Marseille, Marseille, France; 3 INSERM, Marseille, France; 4 Institut Paoli-Calmettes, Marseille, France

## 

Stable microtubules organized on the basis of a nine-fold rotational symmetry constitute the structural basis of centrioles and basal bodies. A number of proteins associate with these structures to drive the process of centrosomes and cilia formation and to participate in their function. Pericentriolar satellites are small round-shaped particles closely associated with microtubules with PCM1 as a major component. To date they have been described only in vertebrates where they participate in the recruitment of proteins to the centrosomes and cilia. We identified the poorly characterized KIAA0753 protein as a direct interactor of FOR20, a pericentriolar satellite protein required for basal body integrity and ciliogenesis. This binary association allows the recruitment of OFD1, a centriole distal-end protein and regulator of centriole length, to form a ternary complex associated with pericentriolar satellites. Inhibiting expression of KIAA0753 by small inhibitory RNA limits the recruitment of FOR20 and OFD1 onto pericentriolar satellites and centrosomes, and decreases cilia length in serum-starved RPE1 cells. We also show that KIAA0753 has a microtubule-stabilizing activity. We propose that pericentriolar satellite and associated proteins such as PCM1 and KIAA0753 appeared in the course of early metazoan evolution to regulate ancestral eukaryotic centriole/basal body proteins such as FOR20 and OFD1.

